# Aging promotes an increase in mitochondrial fragmentation in astrocytes

**DOI:** 10.3389/fncel.2024.1496163

**Published:** 2024-12-05

**Authors:** Ana Paula Bergamo Araujo, Gabriele Vargas, Lívia de Sá Hayashide, Isadora Matias, Cherley Borba Vieira Andrade, Jorge José de Carvalho, Flávia Carvalho Alcantara Gomes, Luan Pereira Diniz

**Affiliations:** ^1^Instituto de Ciências Biomédicas, Universidade Federal do Rio de Janeiro, Rio de Janeiro, Brazil; ^2^Instituto de Biofísica Carlos Chagas Filho, Universidade Federal do Rio de Janeiro, Rio de Janeiro, Brazil; ^3^Departamento de Histologia e Embriologia, Instituto de Biologia Roberto Alcantara Gomes, Universidade do Estado do Rio de Janeiro, Rio de Janeiro, Brazil

**Keywords:** brain aging, mitochondrial dysfunction, astrocytes, mitochondrial fragmentation, mitochondrial biogenesis and neurodegeneration

## Abstract

**Introduction:**

Brain aging involves a complex interplay of cellular and molecular changes, including metabolic alterations and the accumulation of senescent cells. These changes frequently manifest as dysregulation in glucose metabolism and mitochondrial function, leading to reduced energy production, increased oxidative stress, and mitochondrial dysfunction—key contributors to age-related neurodegenerative diseases.

**Methods:**

We conducted experiments on two models: young (3–4 months) and aged (over 18 months) mice, as well as cultures of senescent and control mouse astrocytes. Mitochondrial content and biogenesis were analyzed in astrocytes and neurons from aged and young animals. Cultured senescent astrocytes were examined for mitochondrial membrane potential and fragmentation. Quantitative PCR (qPCR) and immunocytochemistry were used to measure fusion- and fission-related protein levels. Additionally, transmission electron microscopy provided morphological data on mitochondria.

**Results:**

Astrocytes and neurons from aged animals showed a significant reduction in mitochondrial content and a decrease in mitochondrial biogenesis. Senescent astrocytes in culture exhibited lower mitochondrial membrane potential and increased mitochondrial fragmentation. qPCR and immunocytochemistry analyses revealed a 68% increase in fusion-related proteins (mitofusin 1 and 2) and a 10-fold rise in DRP1, a key regulator of mitochondrial fission. Transmission electron microscopy showed reduced perimeter, area, and length-to-diameter ratio of mitochondria in astrocytes from aged mice, supported by elevated DRP1 phosphorylation in astrocytes of the cerebral cortex.

**Discussion:**

Our findings provide novel evidence of increased mitochondrial fragmentation in astrocytes from aged animals. This study sheds light on mechanisms of astrocytic metabolic dysfunction and mitochondrial dysregulation in brain aging, highlighting mitochondrial fragmentation as a potential target for therapeutic interventions in age-related neurodegenerative diseases.

## Introduction

Aging is associated with various metabolic changes in the brain, which can significantly impact cognitive function and overall brain health. As individuals age, there is a decline in metabolic activity within the brain, including alterations in glucose metabolism and mitochondrial function (Peters, 2006). These changes often lead to reduced energy production and increased oxidative stress, which can contribute to the development of neurodegenerative diseases such as Alzheimer’s disease and Parkinson’s disease. Additionally, aging is also linked to disruptions in lipid metabolism, neurotransmitter regulation, and protein homeostasis, further exacerbating the decline in brain function (Teleanu et al., 2022).

Mitochondrial dysfunction plays a pivotal role in the aging process, affecting various aspects of cellular function, including energy metabolism and oxidative stress. As individuals age, mitochondrial function declines, leading to impaired energy production and increased production of reactive oxygen species (ROS) (Stefanatos and Sanz, 2018). This decline in mitochondrial function has profound implications for brain health, as neurons heavily rely on mitochondrial energy metabolism for their proper functioning. Moreover, astrocytes, the most abundant glial cells in the central nervous system, play crucial roles in supporting neuronal function and maintaining brain homeostasis. Emerging evidence suggests that mitochondrial dysfunction in astrocytes contributes to age-related neurodegenerative diseases and cognitive decline (Diniz et al., 2017; Diniz et al., 2019a; Matias et al., 2022; Popov et al., 2023; Diniz et al., 2024a).

Mitochondrial fragmentation, a process characterized by the division of mitochondria into smaller, dysfunctional organelles, has emerged as a significant factor in neurodegenerative diseases (Knott et al., 2008). This fragmentation disrupts mitochondrial dynamics, impairing their function and contributing to cellular dysfunction. In the context of neurodegenerative diseases, such as Alzheimer’s disease, Parkinson’s disease, mitochondrial fragmentation is often observed in affected neurons (Wang et al., 2017; Wang et al., 2020). These fragmented mitochondria are associated with increased oxidative stress, impaired energy production, and compromised cellular viability (Liu et al., 2020). Importantly, astrocytes, as key regulators of brain homeostasis and neuronal synapse formation (Diniz et al., 2012; Diniz et al., 2014; Diniz et al., 2019b), also exhibit mitochondrial fragmentation in neurodegenerative conditions. Dysfunctional astrocytic mitochondria can compromise their ability to provide energy and metabolic support to neurons, exacerbating neuronal dysfunction and contributing to disease progression. Furthermore, dysfunctional astrocytes may release harmful factors, such as pro-inflammatory cytokines and ROS, further exacerbating neuroinflammation and neuronal damage (Joshi et al., 2019). Recently, our group demonstrated that senescent astrocytes exhibit an accumulation of damaged mitochondria, resulting from impaired mitophagy. This finding suggests that the disruption in the mitochondrial quality control system may contribute to cellular dysfunction and neurodegeneration (Diniz et al., 2024a). Understanding the intricate interplay between mitochondrial dysfunction, aging, and astrocyte function is essential for developing therapeutic strategies to promote healthy brain aging and mitigate the risk of neurodegenerative disorders.

Here, we investigate the role of astrocytic mitochondrial fragmentation in age-related brain decline. Cultured senescent astrocytes displayed a significant increase in the density of smaller mitochondria, a hallmark of fragmentation. This finding coincided with elevated levels of DRP1, a protein known to promote mitochondrial fission. Notably, we observed a similar pattern in the brains of aged animals, with increased DRP1 expression accompanied by a rise in the abundance of smaller mitochondria within astrocytic cytoplasm. This strong correlation between DRP1 expression and fragmented mitochondria in astrocytes of aged animals suggests a crucial role for mitochondrial dynamics in the development of age-related neuropathology. These findings underscore the dynamic nature of mitochondria as a potential therapeutic target for interventions focused on preserving mitochondrial function in astrocytes and, ultimately, combating age-related neurodegeneration.

## Materials and methods

### Animals

Newborn (P0) Swiss mice were used for astrocytes cultures. For *in vivo* experiments, we used male C57Bl/6 mice divided into two groups: 2–3 months-old (young group) and 18–24 months old (aged group). All animals were housed at standard conditions with *ad libitum* access to food and water. Animal handling and experimental procedures were previously approved by the Animal Use Ethics Committee of the Federal University of Rio de Janeiro (CEUA-UFRJ, approval protocol 119/23).

### Primary cortical astrocyte cultures and induction of senescence

Primary cortical astrocyte cultures were established from newborn Swiss mice following previously described methods (Matias et al., 2022; Diniz et al., 2024a). Briefly, cerebral cortices were isolated, and meninges were meticulously removed. The tissues were then incubated in Dulbecco’s Modified Eagle Medium supplemented with F12 nutrient mixture (DMEM/F12; Thermo Fisher Scientific) and 10% fetal bovine serum (FBS; Thermo Fisher Scientific) for approximately 7 days *in vitro* (DIV) at 37°C in a humidified incubator with 5% CO_2_ and 95% air until confluent. Cells were subsequently divided into two groups: control and senescent astrocytes. For the control group, confluent astrocyte cultures were treated with 10 μM cytosine arabinoside (Ara C; Sigma) in DMEM/F12 with 10% FBS for 48 h. Following this, the cells were washed and maintained in DMEM/F12 without FBS for an additional 24 h before fixation or RNA extraction. In contrast, the senescent astrocyte group was washed after Ara C treatment and maintained in DMEM/F12 supplemented with 10% FBS for 30–35 DIV with media changes every 2 days. Twenty-four hours prior to fixation or RNA extraction, senescent astrocyte cultures were incubated in DMEM/F12 without FBS.

### Immunocytochemistry

Astrocytes cultures were fixed with 4% paraformaldehyde (PFA) in phosphate-buffered saline (PBS) at pH 7.4 for 15 min. To block non-specific binding sites, the cultures were then incubated with a solution of 3% bovine serum albumin (BSA), 5% normal goat serum, and 0.2% Triton X-100 in PBS for 1 h at room temperature. Following blocking, the cells were incubated overnight with primary antibodies: rabbit anti-DRP1 (1:100 dilution; Cell Signaling, Cat. #8570) and mouse anti-Mitofusin1 + Mitofusin2 (1:300 dilution; Abcam, Cat. ab57602). After washing with PBS, secondary antibodies (Alexa Fluor 546 or 488-conjugated goat anti-rabbit/mouse IgG; Thermo Fisher Scientific) were applied at appropriate dilutions (1:1,000 or 1:300) for 2 h at room temperature. Finally, nuclei were counterstained with DAPI (Sigma-Aldrich) and the cells were observed using a TE2000 Nikon microscope, a Nexcope NIB-620FL microscope, or a Leica SPE confocal microscope.

### Mitochondrial membrane potential

Primary cultures and senescent astrocytes were seeded in 24-well plates and treated with JC-1 (Thermo Fisher Scientific, Cat. T3168) according to the manufacturer’s instructions. For JC-1 staining, the culture medium was removed, and cells were incubated with 2 μg/mL of JC-1 dye for 30 min at 37°C in the dark. Cells were then washed twice and maintained in Gey’s buffer (NaCl: 137 mM; CaCl_2_: 1.53 mM KCl: 4.96 mM; MgCl_2_: 1.03 mM; KH_2_PO_4_: 0.22 mM; Na_2_HPO_4_: 0.85 mM; MgSO_4_: 0.28 mM; NaHCO_3_: 2.70 mM and C_6_H_12_O_6_: 41.64 mM). The cells were imaged by acquisition of 5 fields at a 20X magnification. The intensity of the red channel and the green channel was normalized by the number of nuclei counted per field, thereby obtaining the average intensity per cell. Subsequently, the average intensity per cell of the red channel was divided by the average intensity of the green channel, thus obtaining the mitochondrial membrane potential. When mitochondrial membrane potential is intact, JC-1 forms aggregates, resulting in red fluorescence. Conversely, when membrane potential is lost, JC-1 remains in its monomeric state, emitting green fluorescence. Therefore, a higher red/green fluorescence ratio indicates a greater proportion of healthy mitochondria within a cell population (Sivandzade et al., 2019).

### Mitotracker staining

Control and senescent astrocyte primary cultures were exposed to 100 nM of MitoTracker™ Red CMXRos (Thermofisher Catalog M7512) for 30 min. Following this, the cells were fixed using 4% PFA for 15 min, and the slides were subsequently stained with DAPI before being examined under a fluorescence microscope.

#### Immunofluorescence analysis

Densitometry for the immunohistochemistry and immunocytochemistry images was performed using integrated density values generated with the ImageJ program (National Institutes of Health, USA, RRID:SCR_003070) or through analysis of the intensity in Leica Application Suite X software (RRID:SCR_013673). Immunocytochemistry data were collected from at least 10 fields per coverslip. The integrated density value was divided by the number of cells in each field. Analyses were performed in duplicate, and the graphs represent the average from at least three independent experiments. Mitochondrial morphology was analyzed using the mitochondrial macro morphology in ImageJ, as described previously (Dagda et al., 2009; Wiemerslage and Lee, 2016). Mitochondria were labeled with MitoTracker, and the macro outlined mitochondrial contours using “analyze particles.” Data on mitochondrial count were extracted and divided by the number of cells in the field to obtain mitochondrial density per cell. Additionally, we assessed the average size of labeled particles, indicative of the average mitochondrial size. The utilization of these parameters has been previously validated in the literature using well-characterized mediators of mitochondrial fission and fusion.

#### Quantitative RT-PCR (qPCR)

The cortical astrocytes were lysed with TRIzol^®^ (Invitrogen), and total RNA was isolated and purified using the Direct-zol™ MiniPrep Plus kit (Zymo Research, Irvine, CA, USA) following the manufacturer’s protocol. The RNA was quantified using a NanoDrop ND-1000 spectrophotometer (Thermo Fisher Scientific, Waltham, MA, USA). For reverse transcription, 1–2 μg of total RNA was used with the GoScriptTM Reverse Transcriptase cDNA reverse transcription kit according to the manufacturer’s instructions (Promega Corporation, an affiliate of Promega Biotecnologia do Brasil, Ltda). Primers were designed and synthesized by IDT-DNA (San Diego, CA, USA). The specific forward and reverse oligonucleotides used were as follows: Ppargc1α: (F) GAATCAAGCCACTACAGCACCG, (R) CATCCC TCTTGAGCCTTTCGTG; TFAM: (F) GAGGCAAAGGATGATT CGGCTC, (R) CGAATCCTATCATCTTTAGCAAGC; TFB1: (F) CTGGTGGTTGAAAAGGACACTCG, (R) CCACTGTCTTCTAA TGTTGCCTG; ATPS5A1: (F) TGGTGAAGAGACTGACGGATGC, (R) TCAAAGCGTGCTTGCCGTTGTC TOMM20: (F) GCTA AGGAGAGAGCTGGGCTTT, (R) TGGTCCACACCCTTCT CGTAGT; VDAC1: (F) AGTGACCCAGAGCAACTTCGCA, (R) CAGGCGAGATTGACAGCAGTCT; MFN1: (F) TCTCCAAGCC CAACATCTTCA, (R) ACTCCGGCTCCGAAGCA; MFN2: (F) GGGGCCTACATCCAAGAGAG, (R) GGAGAACTTTGTCCC AGAGC OPA1: (F TGGGCTGCAGA)GGATGG, (R) CCTGA TGTCACGGTGTTGAT; DRP1: (F) AGACGCTTAATC TGACGTTTGAC, (R) AGGTGGCCTTAACACTATTGACA; Fis1: (F) GAGAAGATCCTCGGGTGCAG, (R) CTTTGGGCA ACAGCTCCTCC; MFF: (F) GTCCCAGAGAGGATCGTCGT, (R) TGCTCGGCTCTCTTCGCTTT and the reference gene RPLP0: (F) CAGGTGTTTGACAACGGCAGCATT, (R) ACT CAGTCTCCACAGACAATGCCA Quantitative real-time PCR was performed using the Fast SYBR Green Master Mix qPCR Master Mix (Applied Biosystems TM) with the following cycling conditions: 95°C for 20 s, followed by 40 cycles of 95°C for 1 s and 60°C for 20 s, using the Quant Studio 7 Flex System (Applied Biosystems TM). The relative expression levels of the genes were calculated using the 2-ΔΔCT method (Livak and Schmittgen, 2001).

#### Transmission electronic microscopy

Tissue fragments were processed and analyzed quantitatively. First, they were fixed in 0.1 M sodium cacodylate buffer (pH 7.2) containing 2.5% glutaraldehyde for 24 h, followed by three washes of 10 min each in the same buffer. Post-fixation was done in 0.1 M sodium cacodylate buffer (pH 7.2) with 1% osmium tetroxide (OsO_4_). The sample was then dehydrated using increasing concentrations of acetone (30, 50, 70, 90, and 100%) and embedded in Poly/Bed812 resin (Ted Pella Inc, Redding, CA, USA). After polymerization, 70 nm ultrathin sections were obtained using an ultramicrotome (Leica Microsystems, USA) and collected on 300 mesh copper grids. The sections were counterstained with 5% uranyl acetate and lead citrate and examined using the JEOL JEM-1011 transmission electron microscope (JEOL, LTD., Akishima, Tokyo, Japan). Digital eletromicrographs were captured using an ORIUS CCD digital camera (Gatan Inc, Pleasanton, California, USA) at magnifications of 10,000× and 50,000×. Individual mitochondria from young and old mice were manually traced using Image J software (NIH, Bethesda, MD, USA), to quantify the following parameters: area (μm^2^), perimeter (μm), and the aspect ratio, which is the ratio of the length of the major axis to the length of the minor axis, as well as the length/diameter ratio of the mitochondria. Mitochondria with a ratio of >2 were considered as long mitochondria (long-mito), as previously described (Zhang et al., 2016; Wen et al., 2022). The mitochondria analyzed were exclusively from cells containing glycogen granules in the cytoplasm, a feature unique to astrocytes in the brain. In electron microscopy images, glycogen appears as electron-dense, spherical granules within astrocytic processes (Phelps, 1972; Vezzoli et al., 2020). Quantitative analysis was performed in five random fields of each glycogen-labeled cell. A total of 326 mitochondria from young animals and 268 mitochondria from elderly animals were analyzed, representing five animals per group.

#### Flow cytometry

Cell dissociation for flow cytometry was adapted from a previously described protocol (Diniz et al., 2024a; Diniz et al., 2024b). Mice were anesthetized with ketamine/xylazine (100 and 10 mg/kg, respectively) and then transcardially perfused with saline. Brains were removed, dissected, and rinsed in PBS. After removing the meninges, the cerebral cortex was dissected into small pieces using a sterile scalpel and centrifuged at 300 × g for 2 min at room temperature (RT), and the supernatant was carefully aspirated. Enzymatic cell dissociation was carried out using 20 U/mL of papain in Gey’s buffer for 20 min at 37°C under agitation. Papain activity was halted with 10% FBS in Gey’s buffer at 4°C. Tissue was mechanically dissociated with a pipette and filtered through a 70 μm nylon cell strainer to remove tissue debris. Cell suspensions were centrifuged at 300 × g for 10 min at 4°C and fixed with 2% paraformaldehyde at 4°C for 2 h. Suspensions were washed three times with PBS followed by centrifugation and then stored in PBS at 4°C. Cell suspensions were centrifuged and permeabilized with saponin (0.1% in PBS) for 15 min at RT, followed by centrifugation and three washes with PBS. Next, the cells were incubated with glycine (1.5 mg/mL) for 15 min at RT and washed three times with PBS. Subsequently, cells were incubated with a blocking solution (PBS/BSA 1%) for 1 h at 37°C, followed by incubation with the primary antibodies: mouse anti-GFAP (1:250; Millipore Cat. MAB360), mouse anti-β-Tubulin III (1:250; Promega, Cat. G7121), rabbit anti-TOMM20 (1:50, Abcam, Cat. ab186735) and rabbit phospho-DRP1 (Ser616) (1:200, Thermo Fisher, Cat. PA5-64821) at 4°C for 24 h. The cell suspension was then centrifuged at 300 × g for 5 min at 4°C, washed three times with PBS, and incubated with the secondary antibodies: Alexa Fluor 488-conjugated goat anti-mouse IgG (1:300; Invitrogen, Cat. A11001) and Alexa Fluor 633-conjugated goat anti-rabbit IgG (1:1,000; Invitrogen, Cat. 21070) at RT for 2 h. After incubation, the cell suspension was centrifuged and washed as described above, and the cells were resuspended in 500 μl of PBS. Background staining for antibodies was determined using antibody non-conjugated cells and fluorochrome-conjugated isotype control cells. Finally, cells were analyzed using a FACS Canto II flow cytometry system (BD Biosciences), and the data were analyzed with FlowJo vX software.

#### Data and statistical analysis

Statistical analysis of the quantitative data was performed using GraphPad software, version 8.0 (GraphPad Software, La Jolla, CA, USA). A confidence interval of 95% was applied, and a *p*-value < 0.05 was considered statistically significant. The data are presented as the mean ± SEM, with the error bars in the graphs indicating the SEM. The *p*-values and sample sizes for all experiments are provided in the figure legends. The Student’s *t*-test was utilized for statistical analyses.

## Results

### Fragmented mitochondria in senescent astrocytes exhibit compromised membrane potential

**FIGURE 1:**
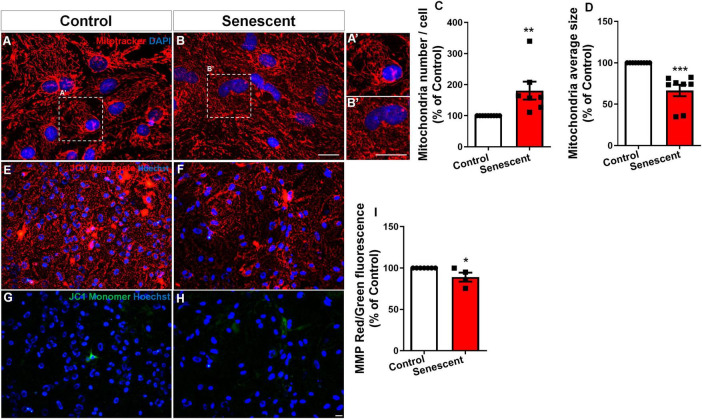
Fragmented mitochondria in senescent astrocytes exhibit compromised membrane potential. Primary murine astrocyte cultures were maintained for 9–10 DIV (control group) or 30–35 DIV (senescent group). Mitochondrial morphology was analyzed by Mitotracker staining **(A,B)**, revealing increased density of mitochondria **(C)** with smaller sizes in senescent cells **(D)**. Mitochondrial membrane potential (MMP) was assessed using JC1 staining, which labeled aggregates in red **(E,F)** and monomers in green **(G,H)** and represented by the ratio between red and green fluorescence **(I)**. Individual data points are plotted and represent individual cultures (*n* = 4–8 cultures/group). Significance was determined using the unpaired Student’s *t*-test. Error bars represent ± SEM. *p < 0.05; **p < 0.01, and ***p < 0.001. Scale bars, 20 μm.

To assess the impact of aging on mitochondrial morphology in astrocytes, we employed an *in vitro* model of senescent astrocytes previously established (Matias et al., 2022; Matias et al., 2023; Diniz et al., 2024a). Both control and senescent astrocytes were stained with Mitotracker Red, a fluorescent dye specific for mitochondria. Subsequent analysis using fluorescence microscopy revealed a significant increase in mitochondrial density and a decrease in the average mitochondrial size in senescent astrocytes compared to controls (Figures 1A–D). Given that fragmentation can compromise mitochondrial function, we next assessed the mitochondrial membrane potential in senescent astrocytes.

The JC-1 assay measures mitochondrial membrane potential (ΔΨm) by comparing red (high ΔΨm) to green (low ΔΨm) fluorescence. The red/green ratio indicates the balance between healthy and dysfunctional mitochondria (Sivandzade et al., 2019). Using the JC-1 probe, we observed a decrease in mitochondrial membrane potential compared to control cultures (Figures 1E–I). These findings suggest that aging promotes mitochondrial fragmentation and impairs mitochondrial function in astrocytes cultures.

### Senescent astrocytes exhibit an imbalance in the mitochondrial fusion and fission pathways

**FIGURE 2:**
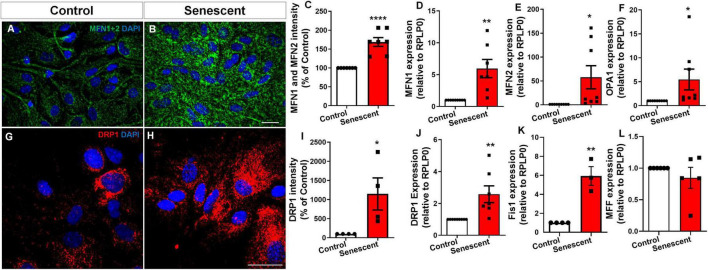
Senescent astrocytes exhibit an imbalance in the mitochondrial fusion and fission pathways. Primary murine astrocyte cultures were maintained for 9–10 DIV (control group) or 30–35 DIV (senescent group). Following this period, protein levels implicated in fusion **(A–F)** and fission **(G–L)** processes were assessed via immunocytochemistry for Mitofusin I and II **(A–C)** and dynamin-related protein-1 (DRP1) **(G–I)**, respectively. Expression of genes involved in mitochondrial fusion, MFN1 **(D)**, MFN2 **(E)**, and OPA1 **(F)**, and for fission, DRP1 **(J)**, Fis1 **(K)**, and MFF **(L)** was performed. Individual data points are depicted, representing distinct cultures (*n* = 4–8 cultures/group). Statistical significance was determined utilizing the unpaired Student’s *t*-test. Error bars represent ± SEM. *p < 0.05; **p < 0.01; ****p < 0.0001. RPLP0: ribosomal protein lateral stalk subunit P0. Scale bars, 20 μm.

Mitochondrial dynamics are regulated by two key processes: fusion and fission. Fusion is controlled by Mitofusin 1 (MFN1) and Mitofusin 2 (MFN2), which are located on the outer mitochondrial membrane, and by Optic Atrophy 1 (OPA1), located on the inner mitochondrial membrane. These proteins work together to merge mitochondria, promoting the exchange of mitochondrial content and maintaining mitochondrial function. Fission is regulated primarily by Dynamin-Related Protein 1 (DRP1), a cytosolic protein that is recruited to the mitochondrial surface by receptor proteins like Fission 1 (Fis1) and Mitochondrial Fission Factor (MFF). DRP1 constricts and divides mitochondria, a process that is important for mitochondrial distribution, removal of damaged mitochondria, and cellular health disruptions in this process are implicated in neurodegeneration (Bertholet et al., 2016). We investigated protein levels associated with mitochondrial fusion and fission in senescent astrocytes. Unexpectedly, we detected a 68.7% increase in the levels of MFN1 and MFN2 (proteins that promote fusion) in senescent astrocytes, as evidenced by both immunocytochemistry (Figures 2A–C) and qPCR (Figures 2D, E). Additionally, we observed a 5-fold increase in OPA1, another fusion protein (Figure 2F). Intriguingly, we observed an 11.5-fold increase in DRP1 staining, a key mediator of mitochondrial fission. (Figures 2G–I). This was further supported by a 2.5-fold rise in DRP1 expression (Figure 2J) and a 5-fold increase in Fis1 expression in senescent cells (Figure 2K). Interestingly, there was no change in MFF expression (Figure 2L). These findings suggest a potential imbalance in the regulatory proteins governing mitochondrial dynamics within senescent astrocytes, with a tilt toward fission despite the upregulation of fusion proteins.

### Aging in mice leads to a decrease in mitochondrial content in both astrocytes and neurons of the cerebral cortex

**FIGURE 3:**
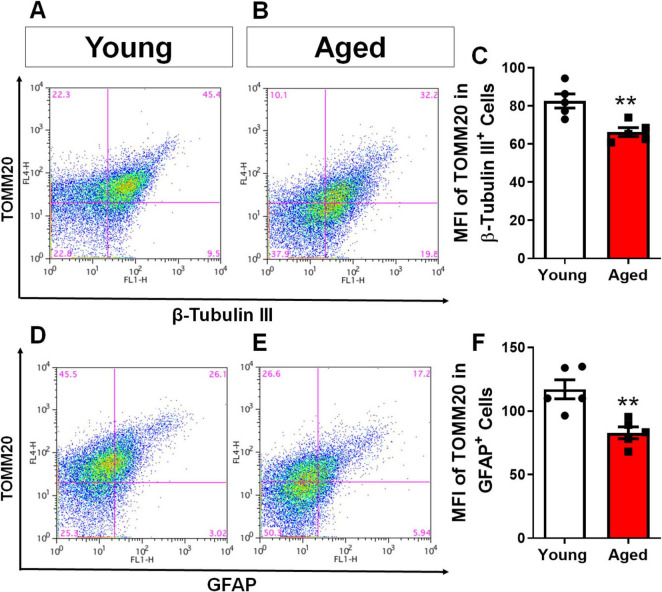
Aging in mice leads to a decrease in mitochondrial content in both astrocytes and neurons of the cerebral cortex. Brain cells from the cerebral cortex of young and aged mice were dissociated and subjected to evaluation of Mean Fluorescence Intensity (MFI) by flow cytometry. The MFI of TOMM20 was analyzed in cells positive for β-Tubulin III, representing the neuronal population **(A–C)**, and TOMM20 in cells positive for GFAP, representing the astrocytic population **(D–F)**. Individual data points are depicted, representing distinct animals analyzed. Statistical significance was determined using the unpaired Student’s *t*-test. Error bars indicate ± SEM. **p < 0.01.

Given the detrimental effects of aging on cellular health, particularly in relation to mitochondrial damage, we investigated mitochondrial density in cortical astrocytes and neurons. Cells were isolated from the cerebral cortex of young and elderly animals. To differentiate cell types and stain mitochondria, we used immunofluorescence labeling with TOMM20 (Translocase of the Outer Mitochondrial Membrane 20), a key protein involved in mitochondrial protein import and commonly used as a marker for mitochondrial mass and distribution, was employed. Astrocytes were labeled with GFAP and TOMM20, while neurons were labeled with β-Tubulin III and TOMM20. We then used flow cytometry to quantify mitochondrial content in both cell populations. Our analysis revealed a significant decrease in the mean fluorescence intensity of TOMM20 in both neurons (Figures 3A–C) and astrocytes (Figures 3D–F) from elderly animals compared to young controls. This indicates a global decline in mitochondrial content within the cerebral cortex with aging.

### Aging impairs mitochondrial biogenesis in the cerebral cortex

**FIGURE 4:**
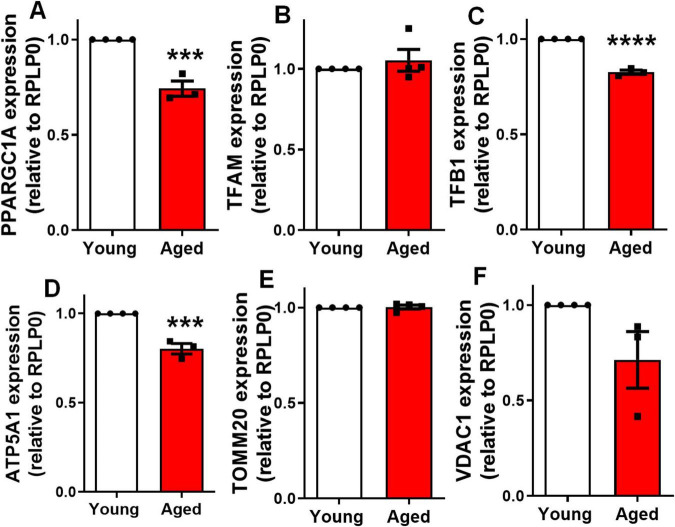
Aging impairs mitochondrial biogenesis in the cerebral cortex. Expression of genes involved in mitochondrial biogenesis was evaluated by qPCR: PPARGC1A **(A)**, TFAM **(B)**, TFB1 **(C)**, ATP5A1 **(D)**, TOMM20 **(E)**, and VDAC1 **(F)** in the cerebral cortex of 2–3-month-old mice (young) compared to 18–25-month-old mice (aged). Individual data points are plotted and represent individual animals (*n* = 3–4 animals/group). Significance was determined using the unpaired Student’s *t*-test. Error bars represent ± SEM. ***p < 0.001 and ****p < 0.0001. RPLP0, ribosomal protein lateral stalk subunit P0.

Mitochondrial biogenesis is the process by which cells increase mitochondrial mass and function, primarily regulated by the Peroxisome Proliferator-Activated Receptor Gamma Coactivator 1 Alpha (PGC-1α). PGC-1α activates transcription factors such as TFAM (Transcription Factor A) and TFB1 (Transcription Factor B1), which drive the replication of mitochondrial DNA and the expression of mitochondrial proteins. Key mitochondrial protein-coding genes, including ATP synthase subunit alpha (ATP5A1), TOMM20, and voltage-dependent anion channel 1 (VDAC1), play essential roles in ATP production and maintaining mitochondrial membrane integrity (Halling and Pilegaard, 2020). Since mitochondrial biogenesis is essential for maintaining mitochondrial density, we employed qPCR assays to analyze the expression of key regulatory genes for these processes. We focused on genes critical for mitochondrial biogenesis, including: PGC1α (PPARGC1A gene), TFAM, TFB1, ATP5A1, TOMM20 and VDAC1. Notably, our findings revealed a significant decrease in the expression of PGC1α (Figure 4A), TFB1 (Figure 4C), and ATP5A1 (Figure 4D), in the cortex of elderly animals compared to controls. No difference was observed in the expression level of TFAM (Figure 4B), TOMM20 (Figure 4E) and VDAC1 (Figure 4F). This suggests a reduction in mitochondrial biogenesis within the cerebral cortex of aged mice.

### Cerebral cortex astrocytes exhibit age-related mitochondrial fragmentation associated with increase of DRP1

**FIGURE 5:**
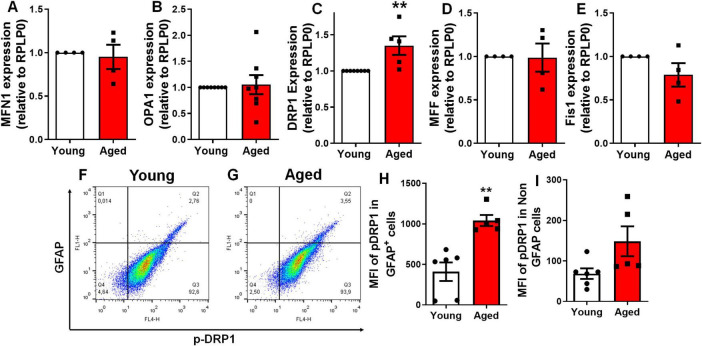
Aging increases DRP1 expression and phosphorylation in the murine cerebral cortex. Expression of genes involved in mitochondrial fusion **(A,B)** and fission **(C–E)** was assessed via qPCR: MFN1 **(A)**, OPA1 **(B)**, DRP1 **(C)**, MFF **(D)**, and Fis1 **(E)**, in the cerebral cortex of 2–3-month-old mice (young) compared to 18–25-month-old mice (aged). Brain cells from the cerebral cortex of young and aged mice were dissociated and analyzed for Mean Fluorescence Intensity (MFI) by flow cytometry **(F,G)**. The MFI of p-DRP1 at Serine 616 was assessed in GFAP-positive cells **(H)**, representing the astrocyte population, and in GFAP-negative cells **(I)**, representing the non-astrocyte population. Individual data points are plotted, representing individual animals (*n* = 3–8 animals/group). Significance was determined using the unpaired Student’s *t*-test. Error bars indicate ± SEM. **p < 0.010. RPLP0, ribosomal protein lateral stalk subunit P0.

To further explore the mechanisms underlying mitochondrial fragmentation *in vivo*, we investigated the expression levels of genes and proteins involved in mitochondrial dynamics within the cerebral cortex of aged mice (Figure 5). Using qPCR, we observed a 34% increase in the expression of DRP1, while no changes were detected in the expression of key fusion (MFN1 and OPA1) or fission (MFF and Fis1) genes (Figures 5A–E). It is well established that phosphorylation of DRP1 at the serine 616 (S616) site activates DRP1-mediated mitochondrial fission and its localization on the outer membrane (Taguchi et al., 2007; Bo et al., 2018). We used flow cytometry to quantify the levels of p-DRP1 at the serine 616 site in different cell populations from aged brains. Our analysis revealed a significant increase in the mean fluorescence intensity of p-DRP1 in astrocytes (GFAP-positive cells), while no significant changes were observed in non-astrocytic populations (Figures 5F–I). These findings suggest that aged animals exhibit enhanced mitochondrial fission activation specifically in astrocytes.

**FIGURE 6:**
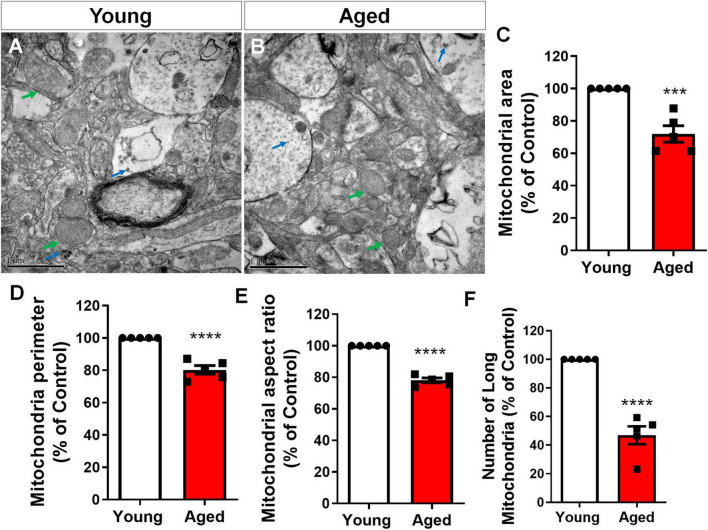
Cerebral cortex astrocytes exhibit age-related mitochondrial fragmentation. Representative Transmission Electron Microscopy (TEM) images of the cerebral cortex of 2–3-month-old mice (young) **(A)** compared to 18–25-month-old mice (aged) **(B)** revealed a decrease in mitochondrial area **(C)**, perimeter **(D)**, aspect ratio **(E)** and number of long mitochondria **(F)**. Astrocytes were identified by the presence of cytoplasmic glycogen granules (blue arrows). Green arrows indicate mitochondria. Individual data points are plotted and represent individual animals (*n* = 5 animals/group). Significance was determined using the unpaired Student’s *t*-test. Error bars represent ± SEM. ***p < 0.001; ****p < 0.0001. Scale bar: 1 μm.

To specifically access mitochondria fragmentation *in vivo*, we performed transmission electron microscopy (TEM) analysis. We identified a striking difference in the mitochondria within astrocytes from elderly mice compared to young controls. Astrocytes were identified based on the presence of unique cytoplasmic glycogen granules, a feature that distinguishes them from other brain cell types. Glycogen granules are exclusive to astrocytes within the central nervous system and serve as an energy reserve, particularly important in regulating neuronal metabolismo (Phelps, 1972; Vezzoli et al., 2020) (Figure 6). Mitochondria from aged brain astrocytes displayed altered morphology, exhibiting a significant reduction in both area (Figure 6C) and perimeter (Figure 6D), as well as lower aspect ratio (Figure 6E). Furthermore, the analysis revealed a significant decrease in the abundance of elongated mitochondria within the aged brain astrocytes (Figure 6F), a hallmark of healthy and functional mitochondria (Rosencrans and Chan, 2023).

Taken together, these results show that astrocytes from the aged brain exhibit increased mitochondrial fragmentation, likely associated with DRP1 activation.

## Discussion

**FIGURE 7:**
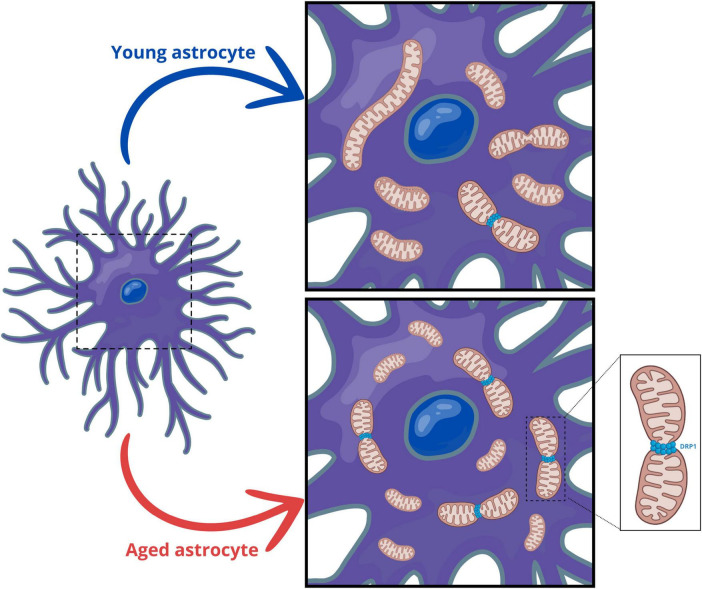
Aging promotes mitochondrial fragmentation in astrocytes. In aged animals, both astrocytes and neurons exhibit a reduction in mitochondrial content, along with decreased mitochondrial biogenesis. Additionally, senescent astrocytes show dysregulation of mitochondrial fusion and fission pathways, resulting in fragmented mitochondria. Consistent with our *in vitro* findings, astrocytes from aged animals display increased activation of DRP1 and fragmented mitochondria.

Astrocytes play a pivotal role in maintaining neurotransmitter balance and recycling neurotransmitters, thereby modulating synaptic transmission and plasticity (Diniz et al., 2020; Zehnder et al., 2021; Zhang et al., 2023). In this context, astrocytic mitochondria provide essential metabolic support to neurons, supplying energy substrates and antioxidants crucial for neuronal survival and function (Zehnder et al., 2021). On the other hand, dysfunctions in astrocytic mitochondria have been reported to be associated with several neurodegenerative diseases (Bang et al., 2019; Xia et al., 2020). However, the mitochondrial morphological and functional changes and their impact on the aging brain remain unclear. Here, we have demonstrated that senescent astrocytes exhibit dysregulation of the pathways controlling mitochondrial fusion and fission, leading to fragmented mitochondria. In aged animals, we also observed a reduction in mitochondrial content in both astrocytes and neurons, accompanied by decreased mitochondrial biogenesis. Supporting our *in vitro* findings, astrocytes from aged animals also displayed fragmented mitochondria (Figure 7).

Mitochondrial dysfunction in senescent astrocytes has been linked to a variety of pathological changes associated with brain aging, including neuroinflammation, neurodegeneration, and cognitive decline (Han et al., 2020). Studies in animal models have demonstrated that mitigating mitochondrial dysfunction in astrocytes can improve cognitive function and delay the progression of neurodegenerative diseases (Gollihue and Norris, 2020; Diniz et al., 2024a).

In this study, we demonstrated that astrocytes and neurons from aged animals exhibit a reduction in mitochondrial content. This finding is supported by a previous study from our group, which observed a decrease in TOMM20 levels and mitochondrial respiration in the cerebral cortex of aged animals (Diniz et al., 2024a). One of the proposed mechanisms responsible for this reduction in mitochondrial content is the decline in mitochondrial biogenesis. While a decline in mitochondrial density in the aged brain has been previously reported, our findings provide the first evidence of a reduction in mitochondrial biogenesis, which may underlie this phenomenon (Navarro and Boveris, 2010). However, it remains unclear whether this decrease in biogenesis occurs in all cell populations, such as astrocytes and neurons. The reduction in mitochondrial biogenesis is demonstrated in neurodegenerative diseases associated with aging, such as Alzheimer’s disease models (Gong et al., 2010; Wang et al., 2021), levels of PGC1α declined in the Alzheimer’s disease human brain and were correlated with the severity of dementia and Aβ pathology (Qin et al., 2009; Katsouri et al., 2011). Activation of PGC-1α in astrocytes, a coactivator of mitochondrial biogenesis, therefore represents a promising therapeutic strategy to improve mitochondrial function and suppress inflammation in the model of multiple sclerosis (Nijland et al., 2014).

However, the exact mechanisms by which mitochondrial dysfunction in senescent astrocytes contributes to brain aging remain under investigation. Recently, we demonstrated that senescent astrocytes exhibit an accumulation of damaged mitochondria due to impaired mitophagy, resulting in increased susceptibility of astrocytes to cell death following a mitochondrial insult (Diniz et al., 2024a). Our group demonstrates that astrocytic synaptogenic capacity and promotion of neurite growth are reduced in senescent astrocytes (Matias et al., 2022). In addition to this finding, primary senescent astrocytes from rats induce loss of mitochondrial membrane potential and alter mitochondrial dynamics in cortical neurons (Morales-Rosales et al., 2021).

In this study, we found that senescent astrocytes exhibited an increase in the quantity and a reduction in the size of mitochondria, which was confirmed by our *in vivo* evidence demonstrating the presence of mitochondria with smaller area and perimeter in astrocytes of elderly mice. These findings suggest an increase in mitochondrial fragmentation imposed by aging. Mitochondrial dynamics, regulated by a balance between fusion and fission processes, are crucial for maintaining mitochondrial homeostasis and cellular functions (Adebayo et al., 2021). Mitochondrial fusion involves the merging of individual mitochondria, mediated by proteins such as MFN1 and MFN2, which tether and fuse the outer mitochondrial membranes. This process facilitates the exchange of contents and promotes the mixing of mitochondrial DNA, contributing to mitochondrial quality control and cellular adaptation to stress (Emery and Ortiz, 2021). Similar to our data, a study conducted in senescent melanoma cells observed an increase in MFN1 and 2 expression and levels, indicating alterations in mitochondrial fusion. However, in the aforementioned study, the mitochondria of senescent cells were larger, and the reduction of DRP1 was only found in the mitochondrial fraction (Martinez et al., 2019).

Evidence from the literature indicates that senescent cells exhibit an increase in oxygen consumption rate (Hutter et al., 2004; Son et al., 2017; Miwa et al., 2022; Bang et al., 2023; Diniz et al., 2024a). Within this context, the modulation of proteins involved in the mitochondrial fusion pathway has recently gained prominence, not only for their role in mitochondrial fusion but also for their impact on increasing metabolism (Li et al., 2020). The increase in oxygen consumption rate in aged normal human fibroblasts is associated with a shift in mitochondrial dynamics toward fusion. Genetic knockdown of MFN1 and OPA1 in old normal human fibroblasts decreased the oxygen consumption rate and shifted metabolism toward glycolysis. Downregulation of MFN1 and OPA1 also suppressed the radiation-induced increase in doubling time of human fibroblasts (Son et al., 2017). A recent publication from our group demonstrated that senescent astrocytes exhibit higher mitochondrial maximum respiration (Diniz et al., 2024a). These findings, along with the upregulation of MFN1, MFN2, and OPA1, support the idea that the modulation of mitochondrial fusion proteins may act as a compensatory mechanism to meet the increased energy demands of senescent cells, despite mitochondrial dysfunction (evidenced by the reduction in membrane potential).

The discrepancy between the increased expression of mitofusin 1, 2, and OPA1 in senescent cortical astrocyte cultures and the unchanged expression of these proteins in the brains of aged animals is intriguing. This discrepancy may be attributed to several factors. First, the *in vitro* environment of astrocyte cultures is highly controlled and simplified compared to the complex *in vivo* microenvironment of the aging brain (Lange et al., 2012). Second, *in vitro* senescence may represent a more advanced stage of mitochondrial dysfunction compared to the aging process *in vivo* (Miwa et al., 2022). Moreover, the cellular heterogeneity in the aging brain, with the coexistence of both senescent and non-senescent astrocytes, may mask specific changes in cellular subpopulations. It is possible that senescent astrocytes *in vivo* exhibit distinct metabolic profiles and, consequently, different regulations of fusion proteins. Finally, compensatory mechanisms or the presence of other cell types in the brain may counterbalance the effects of aging on mitochondrial dynamics *in vivo*. Further studies are needed to fully understand the complex interaction between cellular aging, mitochondrial function, and protein expression in both *in vitro* and *in vivo* models.

Conversely, mitochondrial fission divides mitochondria into smaller fragments and is orchestrated by DRP1, which forms a ring-like structure around mitochondria to constrict and divide them (Taguchi et al., 2007). Additional proteins involved in fission include MFF and Fis1, which recruit DRP1 to the mitochondrial outer membrane (Qin and Xi, 2022). The dynamic interplay between these fusion and fission proteins regulates mitochondrial morphology, distribution, and function, impacting various cellular processes, including energy production, apoptosis, and calcium signaling. Dysregulation of mitochondrial dynamics has been implicated in numerous diseases, highlighting the importance of understanding these processes for developing targeted therapeutic interventions (Qin and Xi, 2022)

We observed that in cultures of senescent astrocytes and in the brains of elderly animals, there is an increase in fragmented mitochondria that correlates with the upregulation of DRP1 expression. Consistent with our findings, in Drosophila ovarian germline stem cells as they age, mitochondrial fragmentation and the expression of the mitochondrial fission regulator, DRP1, are both increased, while mitochondrial membrane potential is reduced. These results demonstrate that mitochondrial dynamics are altered during physiological aging, affecting cell homeostasis via coordinated changes in cell signaling (Amartuvshin et al., 2020).

Mitochondrial fission plays a significant role in maintaining mitochondrial quality control and cellular homeostasis. While essential for cell division and distribution of mitochondria, excessive or dysregulated fission can lead to fragmentation of mitochondria, impairing their function. Dysfunctional mitochondrial dynamics, as observed here, particularly excessive mitochondrial fission, have been associated with oxidative stress, mitochondrial DNA damage, and cellular senescence in various cell types (Twig et al., 2008; Miwa et al., 2022). Fragmented mitochondria are often associated with decreased ATP production and compromised membrane potential, ultimately contributing to cellular dysfunction and pathological conditions (Motori et al., 2013; Joshi et al., 2019; Batista et al., 2021). Although positive regulation of DRP1 in midlife extends the lifespan and health of Drosophila by inducing mitochondrial fission and reducing the accumulation of dysfunctional mitochondria in aged flight muscle (Rana et al., 2017), the anti-aging effects of DRP1 require functional mitophagy, which is typically compromised in the aging brain (Caponio et al., 2022).

On the other hand, impaired fusion events can result in the accumulation of damaged mitochondria and the inability to eliminate dysfunctional organelles through mitophagy, further exacerbating the senescent phenotype. Therefore, the dysregulation of mitochondrial fusion and fission pathways in senescent astrocytes likely contributes to mitochondrial dysfunction, oxidative stress, and the overall senescence-associated phenotype observed in these cells (Ma et al., 2020; Kleele et al., 2021; Miwa et al., 2022).

With advancing age, alterations in mitochondrial dynamics occur in the brain, characterized by increased mitochondrial fragmentation and impaired mitochondrial fusion. These changes contribute to mitochondrial dysfunction, synaptic loss, and neuronal degeneration, all of which are hallmark features of age-related neurodegenerative diseases such as Alzheimer’s and Parkinson’s disease (Joshi et al., 2019; Bantle et al., 2020). Understanding the impact of mitochondrial dynamics on brain aging is essential for developing strategies to preserve mitochondrial function and mitigate age-related cognitive decline and neurodegeneration.

## Conclusion

In conclusion, our study demonstrates that aging significantly impacts mitochondrial dynamics and function in the cerebral cortex, with a particular emphasis on astrocytes. Senescent astrocytes exhibit fragmented mitochondria with compromised membrane potential, reflecting an imbalance in the mitochondrial fusion and fission pathways. Additionally, aging in mice is associated with a marked decrease in mitochondrial content in both astrocytes and neurons of the cerebral cortex, suggesting a broad mitochondrial decline across cell types within this brain region. Furthermore, we observed an impairment in mitochondrial biogenesis and an increase in DRP1 expression and phosphorylation in the aged cerebral cortex, both of which contribute to the observed age-related mitochondrial fragmentation in astrocytes. These findings provide insights into the cellular mechanisms underlying age-related mitochondrial dysfunction and highlight the role of astrocytes in maintaining brain health during aging, offering potential avenues for therapeutic interventions aimed at preserving mitochondrial integrity in the aging brain.

## Data Availability

The original contributions presented in this study are included in this article/supplementary material, further inquiries can be directed to the corresponding author.
